# 5-Fluorouracil Induce the Expression of TLR4 on HCT116 Colorectal Cancer Cell Line Expressing Different Variants of TLR4

**Published:** 2013

**Authors:** Homa Davoodi, Seyed Reza Hashemi, Heng Fong Seow

**Affiliations:** a *Golestan University of Medical Sciences, Gorgan, Iran. *; b *Faculty of Animal Sciences, Gorgan University of Agricultural Sciences and Natural Resources, Gorgan, Iran.*; c *Immunology Unit, Department of Pathology, Putra Malaysia University, 43400, Serdang Selangor, Malaysia. *

**Keywords:** TLR4, Polymorphisms, 5-FU, Colorectal cancer, Chemotherapy

## Abstract

Two common single nucleotide polymorphisms (SNPs) of the human TLR4 gene, namely Asp299Gly (D299G) and Thr399Ile (T399I), have been shown to impair the ability of certain individuals to respond properly to TLR4 ligands. 5-Fluorouracil (5-FU) is widely used for the treatment of patients with advanced colon cancers. The present study examined the impact of two common polymorphisms of the TLR4 genes on the response of the HCT116 colorectal cancer cells to 5-FU. HCT116 was transfected with Flag-CMV1-TLR4 wild-type (WT) and D299G, T399I expression plasmids. The cytotoxic effect of 5-FU on transfected cells was assessed by MTT assay. FACS analysis was performed to show the effect of 5-FU and LPS on the expression of different variants of TLR4. The lowest IC_50_-value was measured in cells expressing the WT TLR4 and non-transfected cells were more resistance to the drug compared to the other cells. 5-FU significantly induced the expression of TLR4 protein in the presence and absence of LPS. 5-FU also induced HMGB1 secretion, Cas3 and PARP activity and these effects were stronger in cells expressing WT TLR4 than the other cells. In conclusion, 5-FU-induced TLR4 expression and LPS had synergistic effect with 5-FU to induced apoptosis in colorectal cancer cells.

## Introduction

Toll-like receptors (*TLRs*) are the most important receptors in innate immunity that have been identified as a major class of pattern-recognition receptors. The *TLR *family comprises at least eleven members; these *TLRs *recognize a limited but highly conserved set of molecular structures (1, 2). *TLR4*, considered one of the most important TLRs, recognizes lipopolysaccharide (LPS) of Gram-negative bacteria. In addition to the LPS*, TLR4 *recognizes heparan sulfate, heat shock proteins, and High Mobility Group Box1 (HMGB1) among other endogenous substances (3). It has been reported that the ability of certain individuals to respond properly to *TLR4 *ligands may be impaired by single-nucleotide polymorphisms (SNPs) within *TLR4 *genes (4-6). Two cosegregating single nucleotide polymorphisms (SNPs) of the human *TLR4 *gene, namely Asp299Gly (rs4986790) and Thr399Ile (rs4986791), have been correlated with a hyporesponsiveness to inhaled LPS (7). The identification of *TLR4 *Asp299Gly and Thr399Ile mutations might be important for the individual risk assessment of cancer patients treated by chemotherapy (8, 9). Human colon cancers show heterogeneous behavior at the molecular and cellular levels, and the tumor cells can develop resistance to chemotherapy. 5-Fluorouracil (5-FU) is widely used for the treatment of patients with advanced colon cancers and is still the mainstay of chemotherapy (10). However, this anticancer effect varies widely among individual patients. Some patients suffer adverse effects of 5-FU chemotherapy, while others do not. Therefore, the identification of the novel biological markers of 5-FU-resistant in tumor cells is one of the most important steps of developing optimal treatment strategies (11, 12). Microbes or microbial products possess potent anticancer action (13, 14). LPS, “a major component of the outer membrane of Gram-negative bacteria” is among the most potent TLR4 agonists which may affect the response to 5-FU chemotherapy in colorectal carcinoma. The Asp299Gly polymorphism with an allelic frequency of approximately 6% in individuals of mixed European descent (1, 7) was found to prevent the binding of HMGB1 to *TLR4 *in a dominant negative fashion (15). The genotoxic agents that activate the Poly (ADP-ribose) polymerase (PARP), including alkylating agents, can stimulate the release of HMGB1 from its association with chromatin, most likely as a result of the direct PARP-mediated poly adenosyl ribosylation of HMGB1 (16-18). Chemotherapy-induced cell death triggers the release of the high-mobility group box 1 protein (HMGB1), which stimulates *TLR4 *and elicits an immune response that is required for the success of the therapy (4). One of the major goals of oncology is to predict the response of patients with cancer to chemotherapeutic agents by employing laboratory methods variously called ‹tumor chemosensitivity assays›, ‹drug response assays› or ‹drug sensitivity assays› *in-vitro *(19, 20). The present study evaluated the impact of two common single nucleotide polymorphisms (SNPs) of the human *TLR4 *gene on the chemosensitivity of colon cancer cells to 5-fluorouracil. 

*Cell lines and reagents*

A number of studies have revealed that HCT116 ‘colorectal carcinoma cell line’ do not express detectable amount of TLR4 (21, 22). Therefore, HCT116 cells were chosen for transfection to eliminate the basal effects of TLR4 of the cells. HCT116 (ATCC, CCL-247) was obtained from ATCC and maintained in RPMI medium supplemented with 10% fetal bovine serum and 0.6% Pen-Strep at 37°C in a 5% CO_2_ atmosphere (All cell culture reagents were purchased from Invitrogen, Carlsbad, CA, USA). LPS (*Escherichia coli *0111:B4) was purchased from Sigma-Aldrich and 5-FU was from Choongwae Pharma Corporation (Seoul, Korea). The levels of HMGB1 in the culture medium or cell lysate were analyzed by Western Blotting using rabbit monoclonal Ab (Abcam). Antihuman *TLR4 *antibody against the surface *TLR4 *was obtained from eBioscience. The apoptosis sampler kit containing rabbit-derived polyclonal antibodies (Abs) against caspase-3, PARP and the corresponding cleaved forms were obtained from Cell Signaling Technology. Apoptosis was determined using a commercial Annexin V-FITC apoptosis detection kit (BD Biosciences). Flag-CMV1-TLR4 wild-type and Flag-CMV1-TLR4 mutants (D299G and T399I) expression vectors were kindly provided as a gift by Prof. Dr. Vogel and Dr. Rallabhandi (University of Maryland). The DNA plasmid pmaxGFP (from Amaxa) was used as a positive control for transfection.

TurboFect™ *in-vitro *Transfection Reagent was from Fermentas.

*MTT assay*

The cells were seeded in a 96-well plate with a density of 5 × 10^3^/well 24 h before the transfection. Three hundred ng of plasmid DNA harboring Wild-type or mutants *TLR4 *was diluted in 100 μL of serum free RPMI medium, 0.6 μL of TurboFect Transfection Reagent (Fermentas) was added to the diluted DNA; the solution was mixed by pipetting. Then, the mixture was incubated for 15-20 min at room temperature. One hundred μL of the TurboFect /DNA mixture was added to each well and then incubated for a further 24 h post-transfection. The transfected cells were then treated with increasing concentrations of 5-FU at 0-625 μg/mL for 24 and 48 h. At the end of each time point, 10 μL of 5 mg/mL MTT [3-(4, 5-dimethylthiazol-2-yl)-2, 5-diphenyltetrazolium bromide] solution was added (final concentration 0.5 mg/mL and stock solution 5 mg/mL MTT in PBS), for 4 h. The MTT solution and medium were removed and 100 μL DMSO was added to each well. Absorbance was measured at 570 nm using the ELISA microplate reader.

*FACS analysis of TLR4 expression and apoptosis in transfected cells treated with 5-FU*

The cells were seeded in a 6-well plate with a density of 5 × 10^5^ cells/well 24 h before the transfection and then transfected as explained above. Transfected cells were then treated with different concentrations of 5-FU for 48 h in the presence and absence of μg/mL LPS for 24 h. The cells were collected and incubated with one μg antihuman *TLR4 *(eBioscience) antibody against the surface *TLR4 *for 30 min on ice in the dark. One mL PBS/ 0.5% BSA was added, centrifuged at 200 *g*, 4°C for 10 min, the supernatant discarded and the above steps were repeated for secondary Ab ( mouse APC-conjugated anti-human IgG Ab). The cell pellet resuspended in pre-diluted binding buffer in a concentration of 1 × 10^6^ cells/mL. Five μL of FITC Annexin V and 5 μL PI was added to 100 μL of solution (1 × 10^5^ cells) then incubated for 15 min at RT (25°C) in the dark. Four hundred μL 1X Binding Buffer was added to each tube then analyzed by flow cytometry. Ten thousand events were collected and analyzed using the CellQuest software (BD Biosciences).

*Western blots (WB) analysis*

Transfected cells were treated with different concentrations of 5-FU for 48 h in the presence and absence of 1 μg/mL LPS. The culture supernatant was removed and frozen at - 80°C for detection of secreted HMGB1. Secreted HMGB1 in culture supernatant was concentrated by acetone (1.8 mL/200 μL supernatant) (23). The level of HMGB1 in the culture medium and Whole-cell lysates were analyzed by western blot (WB) using rabbit monoclonal Ab (Abcam). Caspase-3 activity and Poly (ADP-ribose) polymerase (PARP) cleavage were also analyzed by WB (Rabbit, Cell signaling technology) in cell lysate.

*Statistical analysis*

Statistical analyses were conducted using the ANOVA general linear models procedure (GLM) of SAS software (SAS Institute, 2005). When ANOVA revealed significant effects, means were separated by Duncan’s multiple range tests. Data with p < 0.05 were considered to be significant.

## Results

*MTT assay*

**Figure 1:**
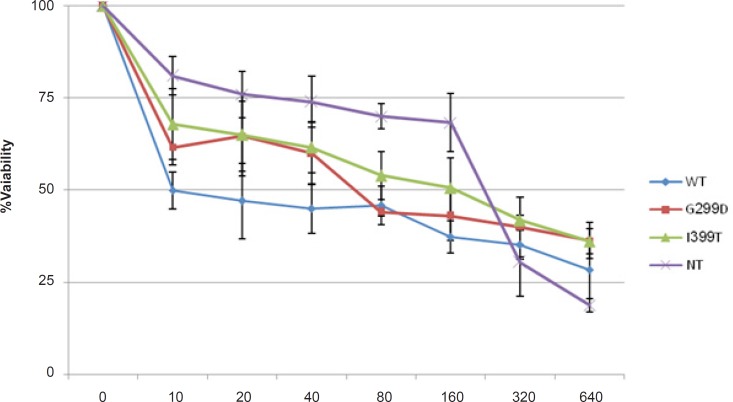
HCT116 cells Transfected with wild-type genotype (WT), D299G, T399I and non-transfected cells (NT) were treated with indicated 5-FU concentration for 48 h. Cell viability was determined using MTT assay. The results represent the mean ± SD of three independent experiments.

MTT assay was performed to evaluate the effect of 5-FU on the survival of cells expressing different variants of TLR4 (Figure 1). 5-fu-treated cells expressing wild type TLR4 showed lower cell viability than the other cells and non-transfected cells were the most resistant cells to 5-fu. The IC_50_-value is shown in Table 1. These results indicated that wild type TLR4 expression made the colorectal cancer cells more sensitive to 5-FU.

**Table 1:** IC_50_ of transfected HCT116 cells treated with 5-FU

**HCT116 cells**	**IC**_50_ **(μg/mL)**
	**48 h**
**WT**	10
**D299G**	60
**T399I**	100
**NT**	200

*Apoptosis assay*

**Figure 2:**
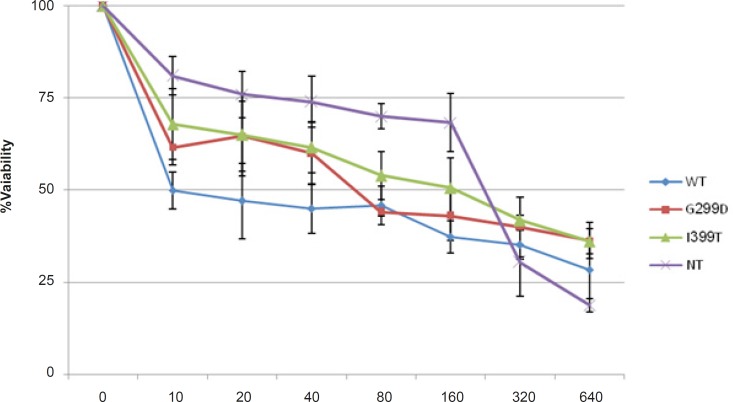
The cells were stained with annexin V and PI subjected to FACS assay of cellular apoptosis. Values are the mean ± SE of three independent experiments; *P < 0.05(Compared with LPS-treated WT cells).

The results of annexin V/PI apoptosis assay showed the higher percentage of apoptosis in cells with WT TLR4 compared to the other cells. LPS had a synergistic effect with 5-FU at a concentration of 120 μg/mL of 5-FU to induce apoptosis (Figure 2). In cells transfected with WT *TLR4 *plasmid, LPS without drug induced the resistance to apoptosis. In contrast, in the presence of drug, LPS induced the apoptosis. However, in mutants and non-transfected cells, LPS induced the apoptosis both in the presence and absence of drug.

*Effect of 5-FU and LPS on the expression of different variants of TLR4*

**Figure 3:**
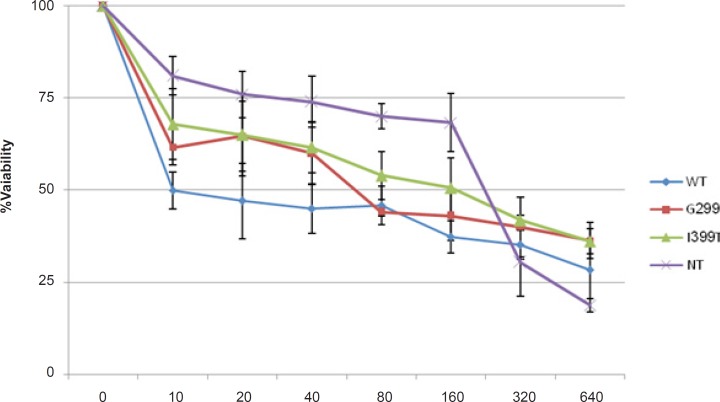
FACS analysis of *TLR4 *expression on transfected HCT116 cells with Wild-type, D299G and T399I genotypes and non-transfected cells (NT) pretreated with different concentrations of 5-FU for 48 h in the absence or presence of LPS (1 μg/mL for 24 h). Values are the mean ± SE of three independent experiments, * p < 0.05(Compared with D299G mutant cells with or without LPS

In order to investigate the effect of 5-FU on the expression of *TLR4 *in colorectal cancer cells and to address the differences between *TLR4 *variants, HCT116 cell line transfected with different *TLR4 *genotypes were treated at different concentrations of 5-FU (0, 125, 625 μg/mL) in the presence and absence of LPS (1 μg/mL for 24 h). As shown in Figure 3, treatment with 5-FU at a concentration of 125 μg/mL significantly increased the expression of *TLR4 *protein as analyzed by FACS in the presence and absence of LPS. LPS without drug also increased the expression of TLR4 on the cells. LPS has a synergistic effect with 5-FU to up-regulate *TLR4 *expression on WT and T399I cells. The level of induced-TLR4 expression was lower in D299G cells compared to the WT and T399I cells (Figure 3). 

*Effect of 5-FU and LPS on HMGB1 release and caspase-3 and PARP cleavage*

**Figure 4:**
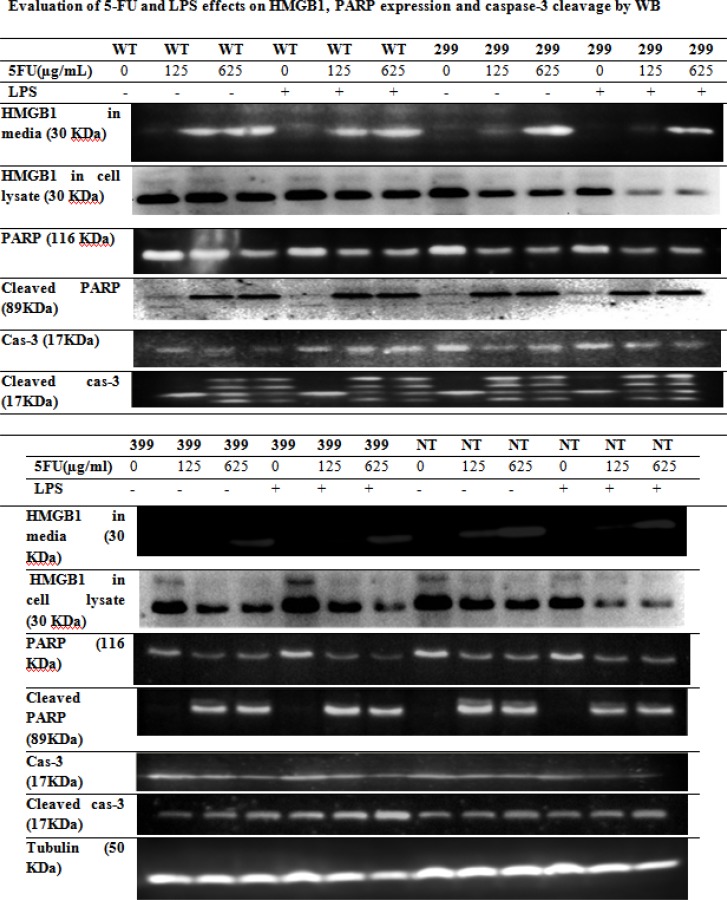
Western blot analysis of HMGB1, PARP expression and caspase-3 cleavage in transfected HCT116 cells with Wild-type (WT), D299G and T399I mutants *TLR4 *were treated with different concentrations of 5-FU for 48 h in the presence or absence of 1 μg/mL LPS for 24 h.

Transfected HCT 116 cells were treated with different concentrations of 5-FU for 48 h in the presence and absence of 1 μg/mL LPS for 24 h. The level of HMGB1 in the culture medium and cell lysate was assayed by Western blotting. The level of HMGB1 release from cells with wild-type TLR4 was higher than HMGB1 release from mutants (Figure 4). HMGB1 release from wild-type and non-transfected cells was detected in media at the concentration of 125 and 625 μg/mL of drug. However, in cells transfected with D299G or T399I variants, HMGB1 release was detected only at 625 μg/mL concentration of drug. We also analyzed the HMGB1 levels in whole-cell lysate by WB. The amount of HMGB1 inside the cells was decreased and caspase-3 and PARP cleavage were increased after the drug treatment.

## Discussion

Patients treated with anticancer chemotherapy have varied response to treatment. Pharmacogenetic and genomic studies can be used to identify the genetic variants that contribute to the individual variation in susceptibility to chemotherapy-induced cytotoxicity (24, 25). The purpose of this study was to investigate the impact of Asp299Gly and Thr399Ile *TLR4 *polymorphisms on Chemosensitivity of HCT-116, Colon Cancer Cells to 5-Fluorouracil. MTT assay was performed to determine the cytotoxic effect of 5-FU on HCT-116 cells expressing different variants of *TLR4*. The results of this study demonstrated, for the first time, that the expression of TLR4 made colon cancer cells more sensitive to 5-Fu. The IC_50_ for non-transfected cells was 200 μg/mL which was the most resistant cells compared to the other cells [IC_50_ for WT = 10 μg/mL, IC_50_ for D299G = 60 μg/mL and for T399I ≈ 100 μg/mL]. In the current study, we also showed that 5-FU activated the PARP which lead to HMGB1 release. The Maximum amount of released HMGB1 was observed in cells with WT *TLR4*. Chemotherapy-induced cell death triggers the release of the HMGB1, which stimulates *TLR4 *and elicits an immune response that is required for the success of the therapy (26). The genotoxic agents that activate the poly-adenosyl- ribosyl polymerase (PARP) can stimulate the release of HMGB1 from its association with chromatin, most likely as a result of the direct PARP-mediated poly adenosyl ribosylation of HMGB1 (16, 27). More interestingly, for the first time we demonstrated that 5-FU treatment significantly induced the expression of *TLR4 *protein on the surface of HCT116 cells in the presence and absence of LPS. LPS had a synergistic effect with 5-FU on up-regulation of TLR4 expression. This is in contrast to a study by Sun *et al*., 2008 which showed that Rapamycin, an immunosuppressant agent which is recently used for cancer therapy, inhibited the expression of TLR4 on colon cancer cells (28). Resistance of non-transfected cells to 5-FU-induced cytotoxicity in our study may be explained by the lower expression of TLR4 and lower amount of HMGB1 released from these cells. On the other hand, wild-type TLR4 genotypes were the most sensitive cells to the 5-FU as these cells secreted more HMGB1 to the media and express more TLR4 in response to the drug and LPS compared to the other cells. According to our results, LPS significantly increased the level of drug induced-apoptosis. Therefore, LPS may be used as an adjuvant with 5-FU to induced apoptosis in colon cancer cells. Wild-type cells treated with LPS only had low number of apoptotic cells; however, apoptosis was increased in the presence of drug and LPS. These results are consistent with those of other studies, which reported that LPS pretreatment induced resistance to the apoptosis in lung and ovarian cancer cells, (29, 30). As a conclusion, TLR4 expression can contribute to the chemosensitivity of colon cancer cells to 5-FU treatment. Our data suggest that LPS enhances 5-FU-induced apoptosis in colon cancer cells. Therefore, LPS may be useful as an adjuvant in 5-FU chemotherapy. We also found that TLR4 expression on colorectal cancer cells was up-regulated by 5-FU treatment. This may contribute to the cytotoxic effect of drug as HCT116 cells with WT TLR4 were more sensitive to the 5-FU than cells with mutant *TLR4*. This may be due to the higher level of HMGB1 release and TLR4 expression induced by 5-FU in cells with WT *TLR4 *variant compared to the other cells. The findings of this study have several important implications for future studies of cancer immunotherapy.
